# Transthyretin cardiac amyloidosis in Saudi Arabia and the Middle East: insights, projected prevalence and practical applications

**DOI:** 10.3389/fcvm.2023.1265681

**Published:** 2023-10-25

**Authors:** Dania Mohty, Mohamed H. Omer, Omar Ahmad, Islam Alayary, Talal Alzahrani, Thibaud Damy, Bahaa Fadel

**Affiliations:** ^1^ Heart Center, King Faisal Specialist Hospital and Research Center, Riyadh, Saudi Arabia; ^2^ College of Medicine, Al Faisal University, Riyadh, Saudi Arabia; ^3^ School of Medicine, Cardiff University, Cardiff, United Kingdom; ^4^ Rare Diseases Medical Affairs, Pfizer Inc., Jeddah, Saudi Arabia; ^5^ Department of Medicine, College of Medicine, Taibah University, Medina, Saudi Arabia; ^6^ Referral Center for Cardiac Amyloidosis, Department of Cardiology, Mondor Amyloidosis Network, GRC Amyloid Research Institute, Clinical Investigation Center 006, DHU A-TVB INSERM U955 all at CHU Henri Mondor, UPEC, Créteil, France

**Keywords:** cardiac amyloidosis, transthyretin, epidemiology, prevalence, incidence, Saudi Arabia, Middle East

## 1. Introduction

Transthyretin (TTR) amyloidosis is a systemic disease characterized by the deposition of insoluble wildtype or mutant TTR fibrils in several tissues. Cardiac involvement results in an infiltrative cardiomyopathy that leads to progressive heart failure and carries a high rate of morbidity and mortality (1, 2). The diagnosis of transthyretin cardiac amyloidosis (ATTR-CA) can be difficult to establish, particularly at an early stage, due to a multitude of reasons. It may initially present with extracardiac symptoms and requires a high index of clinical suspicion together with a certain level of expertise and a multimodality imaging approach to confirm the diagnosis. Moreover, ATTR-CA can mimic other forms of hypertrophic and restrictive cardiomyopathy (3). Studies published over the past decade, mainly in western countries, indicate that ATTR-CA is more common than previously believed and that it is significantly underdiagnosed (4–8). The emergence of novel disease-modifying therapies has led to a global effort to improve the diagnosis and management of ATTR-CA (9).

The prevalence of ATTR-CA in the Middle East, and particularly in the largest country that is Saudi Arabia is significantly underrepresented in the literature. What is clear is that this condition remains underdiagnosed, and the vast majority of patients go undiagnosed. Recent studies conducted in the Middle East and the Gulf region demonstrate significant shortcomings regarding the diagnostic modalities and therapeutic approaches for cardiac amyloidosis, mainly due to a lack of awareness among health care professionals (10, 11). Such underdiagnosis or misdiagnosis of ATTR-CA leads to a delay in instituting disease-modifying therapies and thus has detrimental consequences on patient outcomes.

The objectives of this manuscript are to highlight the magnitude of the under/misdiagnosis of ATTR-CA in Saudi Arabia and neighboring Gulf countries and to explore the barriers that impede the proper diagnosis and management of ATTR-CA in the region.

## 2. Discussion

### 2.1. Assessing the magnitude of the problem: extrapolating the projected prevalence of ATTR-CA in Saudi Arabia

In order to highlight the magnitude of the underdiagnosis of ATTR-CA in the Middle East and Gulf region, we extrapolated the projected prevalence of ATTR-CA in Saudi Arabia. Published epidemiological studies in Saudi Arabia indicate a case burden for chronic heart failure of approximately 455,222 cases, and thus a prevalence of 1%–2% in the general population (12, 13). Within the chronic heart failure patient cohort, data from the Heart Function Assessment Registry Trial in Saudi Arabia (HEARTS) suggest a prevalence of 12.3% for heart failure with preserved ejection fraction (HFpEF) in the country, thus totaling 55,992 patients with HFpEF (14). A recent meta-analysis of global studies estimated a prevalence of 11% for ATTR-CA among patients with HFpEF (15). Based on the combined data, one can estimate a case burden of 6,159 patients with ATTR-CA in Saudi Arabia.

The estimation of ATTR-CA case burden in Saudi Arabia has several shortcomings. First, our approximation does not take into account the gender preponderance of ATTR-CA among males or the influence of age as the disease increases in incidence with age, since the HEART registry did not factor in either parameter. Second, the projected estimation does not include the prevalence of ATTR-CA amongst patients with severe aortic stenosis undergoing transcatheter aortic valve replacement (TAVR). Several studies have previously highlighted an association between ATTR-CA and severe aortic stenosis in elderly patients being relatively common with an estimated prevalence of 8%–15% (16, 17). However, due to the lack of epidemiological data regarding the number of patients with aortic stenosis undergoing TAVR in Saudi Arabia, it is not possible to extrapolate the prevalence of ATTR-CA within this patient population. Nonetheless, this fact suggests that the projected prevalence of ATTR-CA is likely significantly greater than our estimated case burden.

To our knowledge, there are no published studies that have evaluated the incidence or prevalence of cardiac amyloidosis in Saudi Arabia. Furthermore, our search results utilizing Pubmed and Medline databases have not yielded any current population or cohort-based studies relating to ATTR-CA within the region. We hypothesize that the discrepancy in the expected prevalence of ATTR-CA when compared to the literature emerging from the region is likely due to shortcomings in the diagnosis of cardiac amyloidosis rather than lower rates of prevalence.

### 2.2. Addressing the problem: insights and practical applications to tackle the barriers towards the diagnosis and management of cardiac amyloidosis in the Middle East

This section addresses the possible shortcomings and barriers regarding the diagnosis of ATTR-CA and highlights the practical applications to overcome these issues within the region. ATTR-CA is a disease with a high misdiagnosis rate up to 34%–57% of patients (18). Moreover, the disease displays a significant lag in time from symptom onset to diagnosis with a median diagnostic delay of approximately 3.4 years (17).

Two recent physician surveys have investigated the barriers to the diagnosis of cardiac amyloidosis in the Middle East (10, 11). Findings of both studies suggest that one major barrier lies in the lack of physician awareness and knowledge regarding symptoms and clinical findings that should raise suspicion for a diagnosis of ATTR-CA. Therefore, improving physician education and awareness of patient groups at higher likelihood of ATTR-CA based on past medical history, symptoms, and physical findings should lead to a higher rate of correct and timely diagnosis.

Multidisciplinary education campaigns and meetings are essential to familiarize physicians with the clinical, biological, and imaging clues that should prompt further investigations for the disease. Additionally, subgroups of patients such as those with left ventricular hypertrophy, HFpEF, and those with low flow low gradient aortic stenosis requiring TAVR should be identified and further screened for ATTR-CA. A recent scientific statement from the American Heart Association (AHA) has identified clinical and imaging clues that should prompt further investigation(s) for CA (3) can be useful for this purpose. Finally, establishing national or regional referral pathways for patients with potential ATTR-CA represents an important strategy to improve its detection within the region.

Another significant barrier to the diagnosis of ATTR-CA lies in the utilization of appropriate investigative tools. According to guidelines published by the AHA and European Society of Cardiology (ESC), patients with suspected ATTR-CA and without monoclonal gammopathy should undergo screening by cardiac radionuclide scintigraphy imaging (3, 9). However, both surveys from the Middle East highlighted the lack of awareness among physicians regarding the utility of cardiac radionuclide scintigraphy imaging as well as the lack of access to such imaging modalities. Approximately one third of physicians in the surveys were unfamiliar with the role of cardiac radionuclide scintigraphy for the diagnosis of ATTR-CA, whereas 40% of physicians reported the availability of this imaging modality at their institutions (10, 11). An approach to overcome this barrier to the diagnosis of ATTR-CA would be to encourage the establishment of regional centers with expertise in heart failure that are equipped with appropriate imaging modalities. Moreover, a unified multidisciplinary effort is required among specialists in nuclear medicine and cardiac imaging to establish training programs and improve expertise relating to the assessment and interpretation of cardiac radionuclide scintigraphy in line with pre-existing guidelines (3, 4).

Since the hereditary form of ATTR-CA arises from a mutation in the *TTR* gene that requires proper investigation (9), an additional barrier for the optimal evaluation and diagnosis of CA is the lack of data regarding the prevalence of known genetic variants and the presence of novel mutations in the Middle East and Gulf region. Studies from western countries and Japan show significant difference in the prevalence of acquired *TTR* gene mutations causing ATTR-CA (19, 20). Moreover, a recent systematic review and meta-analysis evaluating the clinical outcomes of ATTR-CA revealed significant geographical disparities in research relating to both hereditary and wild-type ATTR, with a paucity of literature emerging from Asia, South America, and Oceania (21). The findings of this study advocate for greater research efforts in this region, particularly in uncovering the frequency of hereditary ATTR due to the poorer prognosis associated with specific genetic variants and the importance of initiating TTR-targeted therapeutics. Interestingly, a recent genetic study conducted in Saudi Arabia established novel findings regarding the prevalence of *TTR* variants in the region (22). Using data mining to evaluate for potentially pathological *TTR* mutations, three *TTR* variants known to be associated with ATTR-CA and three novel variants were identified. However, the data identified in the study requires further clinical correlation.

Based on the above data, collaborative efforts within the region among specialists in cardiology and genetic medicine are required to identify *TTR* variants associated with hereditary ATTR-CA and to detect the individuals carrying these genetic variants who are at risk of developing the disease. Moreover, individuals with confirmed hereditary ATTR-CA should receive counseling and their family members should be offered genetic testing in line with AHA and ESC guidelines. Hence, a multidisciplinary effort is required to provide access to genetic testing sites that are equipped with appropriate resources and are adequately staffed. Additionally, this setup would allow for the conduction of further genomic studies to identify potentially pathogenic *TTR* variants and simultaneous clinical investigations to validate the findings from these genomic studies.

A pertinent approach towards overcoming the underdiagnosis of ATTR-CA in the region is underpinned by the utilization of regional patient registries and the subsequent establishment of nationwide screening programs. The use of national/regional patient registries may facilitate improved awareness of disease prevalence and incidence, enhance collaborative efforts and communication amongst clinical specialists, improve knowledge and awareness of the disease, facilitate the generation of referral and screening pathways, and enhance the cost-effectiveness of managing patients with ATTR-CA (23). Patient registries, particularly in the context of underdiagnosed diseases such as ATTR-CA, represent a critical strategy to minimize variations in clinical practice whilst enhancing and standardizing treatment standards (23, 24). A nationwide registry under the Saudi Heart Association is currently underway with a goal to facilitate the earlier diagnosis of ATTR-CA in the region, highlight shortcomings and areas of improvement in the diagnosis and management of the disease, and facilitate the provision of subsidized funding for novel TTR-targeted therapeutics.

The underdiagnosis of ATTR-CA in the region has resulted in significant shortcomings in the management of the disease. Physician surveys emerging from the region highlighted deficiencies in the awareness of the management modalities for ATTR-CA (10). The recent development of transthyretin targeted therapies has revolutionized the treatment of the disease. Particularly, the transthyretin (TTR) tetramer stabilizer, tafamidis, has demonstrated the ability to improve all-cause mortality and reduce hospitalization in patients with ATTR-CA (25). Moreover, transthyretin protein silencers such as patisiran and inotersen have shown promising outcomes in the treatment of ATTR-CA and are currently approved by the American Food and Drug Administration (FDA) for the treatment of hereditary ATTR with concurrent neuropathy (3). The underdiagnosis of cardiac amyloidosis in the region coupled with the reduced awareness of the disease, hinders the cost-effectiveness of these novel therapies and reduces their utilization and availability. Regional and national collaborations to create appropriate treatment pathways for patients with cardiac amyloidosis should be undertaken to provide patients with evidence-based therapies. National efforts should be undertaken to perform cost-benefit analyses of the novel therapies utilized in ATTR-CA in order to increase access to these medications in the region. Furthermore, optimizing heart failure therapy is of paramount importance in cardiac amyloidosis. Recent studies have demonstrated that conventional heart failure treatment is generally ineffective in CA, and thus optimized heart failure management utilizing medications that have been found to be effective in amyloid cardiomyopathy-induced heart failure is required (26). Therefore, appropriate guidelines and referral pathways should be implemented when managing patients with amyloid cardiomyopathy associated with heart failure.

The application of AHA and ESC guidelines regarding the diagnosis and management of cardiac amyloidosis in the Middle East and Gulf region is likely to lead to significant advances in understanding the epidemiology and clinical features of the disease in the region, while concomitantly improving patient outcomes. However, the application of the aforementioned guidelines may prove challenging due to potential barriers. Table 1 provides a brief discussion of the AHA and ESC recommendations, barriers impeding their utilization within the region, and practical applications to improve their regional implementation.

**Table 1:** Challenges underlying the implementation of international guidelines for the diagnosis and management of transthyretin cardiac amyloidosis in the Middle East and gulf region and strategies to overcome them.

Guideline recommendations	Challenges underlying its use in the Middle East and Gulf region	Practical solutions to overcome barrier
Use of cardiac magnetic resonance imaging and cardiac radionuclide scintigraphy	• Not widely available • Lack of appropriate expertise	• Establish specialized tertiary care centers equipped with appropriate diagnostic modalities • Provide physicians with appropriate training for proper utilization of imaging modalities • Increase collaboration between national and regional centers and provide referral pathways for patients with cardiac amyloidosis
Genetic testing for hereditary transthyretin cardiac amyloidosis	• Lack of genetic testing laboratories and experienced professionals	• Multidisciplinary regional and national effort to establish centralized genetic centers with appropriate equipment, medical geneticists, and cardiac amyloidosis specialists • Collaborative effort between local centers and establishment of regional guidelines to facilitate referral of patients with hereditary ATTR-CA for genetic counseling and testing
Utilizing transthyretin targeting therapies	• Low prevalence of disease and high financial cost hinder access to therapies	• Establishment of specialized tertiary referral centers staffed by specialists in the management of cardiac amyloidosis • Collaboration with national and international colleagues to create national treatment pathways and guidelines • National effort to increase screening and diagnosis of cardiac amyloidosis • Effort to conduct cost-benefit analysis and subsidize financial cost of therapy in the region

## 3. Conclusion

Limited data are available regarding the prevalence, incidence, and clinical features of ATTR-CA in the Middle East and Gulf region. Based on international epidemiological studies, the majority of cases of cardiac amyloidosis in this region remain undiagnosed. Physician surveys conducted in our region identified key areas for improvement, particularly relating to awareness regarding the diagnosis and treatment of ATTR-CA. Collaborative efforts are required to promote disease awareness and improve physician knowledge regarding the epidemiology, clinical features, and management of ATTR-CA. Moreover, appropriately staffed national and regional specialized referral centers equipped with the relevant diagnostic modalities ought to be established within the region. Multidisciplinary teams consisting of clinical experts from various backgrounds should collaborate to develop clinical pathways to identify and investigate patient groups with high suspicion for cardiac amyloidosis. Implementing these measures would improve the diagnosis and management of cardiac amyloidosis in the region, thereby allowing for disease detection at an earlier stage and the utilization of targeted disease-modifying therapies.
